# Monocyte alteration in elderly hip fracture healing: monocyte promising role in bone regeneration

**DOI:** 10.1186/s12979-024-00413-8

**Published:** 2024-02-03

**Authors:** Clement Shema, Yining Lu, Ling Wang, Yingze Zhang

**Affiliations:** 1 Department of Orthopedic Research Center, The Third Hospital of Hebei Medical University, Shijiazhuang, Hebei P.R. China; 2 Department of Orthopedic Surgery, The Third Hospital of Hebei Medical University, Shijiazhuang, China; 3 Department of Orthopedic Oncology, The Third Hospital of Hebei Medical University, Shijiazhuang, Hebei P.R. China

**Keywords:** Hip fracture, Monocyte subsets, Elderly

## Abstract

Individual aged with various change in cell and cellular microenvironments and the skeletal system undergoes physiological changes that affect the process of bone fracture healing. These changes are accompanied by alterations in regulating critical genes involved in this healing process. Unfortunately, the elderly are particularly susceptible to hip bone fractures, which pose a significant burden associated with higher morbidity and mortality rates. A notable change in older adults is the increased expression of activation, adhesion, and migration markers in circulating monocytes. However, there is a decrease in the expression of co-inhibitory molecules. Recently, research evidence has shown that the migration of specific monocyte subsets to the site of hip fracture plays a crucial role in bone resorption and remodeling, especially concerning age-related factors. In this review, we summarize the current knowledge about uniqueness characteristics of monocytes, and their potential regulation and moderation to enhance the healing process of hip fractures. This breakthrough could significantly contribute to the comprehension of aging process at a fundamental aging mechanism through this initiative would represent a crucial stride for diagnosing and treating age related hip fracture.

## Introduction

Aging is a complex process marked by a series of physiological changes that render individuals more susceptible to various age-related diseases. This aging-associated transformation is often accompanied by a systemic inflammatory dysregulation known as “inflammaging” [1]. Furthermore, immune responses in older individuals undergo significant alterations, collectively referred to as “immune senescence,” which can result in heightened vulnerability to infections, reduced vaccine efficacy, increased frailty, and a heightened risk of developing cancers [2].

One of the body systems profoundly affected by aging is the skeletal system, giving rise to conditions such as osteoporosis and osteoarthritis that become increasingly prevalent with age. Additionally, there is a notable upswing in the incidence of bone fractures, which is associated with higher rates of morbidity and mortality [3, 4]. Among these fractures, hip fractures (HF) stand out as particularly concerning due to their severe consequences. HF not only leads to chronic pain and disability but also entails a high morbidity risk, an increased susceptibility to major depression, and a loss of autonomy, and often necessitates institutionalization for individuals who were previously independent [5]. These detrimental outcomes are likely linked to the altered immune responses observed in the elderly population, as aging is known to contribute to immune system decline, affecting both the innate and adaptive arms of immunity [6, 7]. Numerous studies have revealed immunological changes following hip fractures, including functional alterations in neutrophils and inflammatory shifts in conventional monocytes [8, 9]. Moreover, HF prognosis has been associated with elevated levels of pro-inflammatory cytokines such as IL-6 and TNF-α, as well as a few specific biomarkers [10].

Peripheral blood monocytes, a heterogeneous population comprising approximately 10% of peripheral leukocytes in humans, play a pivotal role in both innate and adaptive immunity. They function as phagocytic cells, eliminating pathogens, and also produce cytokines [9, 10]. Understanding the roles and mechanisms of macrophages, which monocytes can differentiate into, in the context of fractures, may provide valuable insights into predicting the timing of surgery for HF patients and mitigating the immunosuppressive effects that contribute to mortality [11]. Given the high prevalence and grim prognosis of HF in the elderly, it becomes imperative to study monocyte changes in the context of HF, particularly concerning their phenotype, function, and their influence on the regulation of innate and adaptive immune compartments. Monocytes are generally considered to be non-proliferative owing to a short lifespan of about 3 days [12]. In addition, the existence of a “proliferative monocyte” population was observed in vitro and this subpopulation was identified as CD14 + monocytes [13]. Intriguingly, a deuterium labeling study has implied that human monocytes can circulate in the bloodstream for as long as 12 days [14]. Thus, monocytes may undergo the process of cellular senescence during this period. Aging affects the cytokine secretion profiles of monocytes following different TLR ligands stimulation. Specifically, monocytes from older adults exhibit a weaker IL-1β and IFN-β response to LPS and influenza A virus treatment, respectively [15, 16]. Monocyte engineering holds promise as a potential prognostic and treatment-oriented approach for HF. In this review we spotlighted the recent studies and age-related impact on hip fracture with monocytes alteration to the healing processes. We scoped on what changes occur in these cells with age, and how this underlies fracture commonly associated with with healing of HF in elderly individuals.

## Biological function of human monocyte

Monocytes are indispensable innate immune cells originating from bone marrow hematopoietic stem cells, and they persistently circulate in the bloodstream throughout an individual’s life. Their multifaceted roles are integral to various physiological processes encompassing bone healing, tissue development, maintenance of tissue equilibrium, host defense, orchestration and resolution of inflammation, and the facilitation of tissue repair [17].

The versatility of monocytes in maintaining immune balance is paramount. When they migrate out of the bloodstream and undergo differentiation, they assume a multitude of immune functions. In states of normalcy or homeostasis, monocytes differentiate into tissue-resident macrophages, thereby contributing to crucial homeostatic functions within tissues. In contrast, when faced with acute inflammatory reactions or the need for antimicrobial defense, monocytes undergo differentiation into inflammatory macrophages, actively amplifying the immune response. Furthermore, monocytes also take on a vital role in the resolution of inflammation and participate in the intricate processes of tissue regeneration [17].

The dynamic and adaptable nature of monocytes allows them to transition seamlessly between these different roles, depending on the ever-changing demands of the immune system and the body’s physiological status. This ability to shift between functions underscores their importance in immune homeostasis and the overall health of an individual.

### Monocytes subsets heterogeneity

Monocytes have traditionally been classified into three distinct populations based on their expression of CD14 and CD16 receptors, which encode the lipopolysaccharide receptor and the low-affinity FCγ receptor, respectively. These categories are as follows: classical monocytes (CD14^hi^CD16^neg^), making up approximately 80–90% of human blood monocytes; intermediate monocytes (CD14^hi^CD16^hi^), accounting for about 2–5%; and nonclassical monocytes (CD14^low^CD16^hi^), constituting the remaining 2–10% [18].

Recent advancements in single-cell studies, including techniques such as flow cytometry, mass cytometry, and single-cell RNA sequencing, have unveiled an even greater degree of heterogeneity among human monocytes [19]. Through genome-wide analyses and cytometric profiling, researchers have identified a range of cell surface markers that are differentially expressed across the three monocyte subsets. These markers include CCR2, CD36, CD64, CD62L, HLA-DR, CX3CR1, SLAN, and CD11c [19]. Moreover, a recent single- cell RNA sequencing study has provided further insights into the heterogeneity among human intermediate monocytes, potentially reflecting different stages of transition between classical and non-classical phenotypes. This study identified the existence of eight monocyte subsets in the peripheral blood of healthy human subjects, including CD61^+^ and CD9^+^ subsets of non-classical monocytes. Interestingly, the CD9^+^ subset was also detected in mice and is likely associated with platelet binding to these monocytes [19].

Ongoing research into monocyte heterogeneity and their roles in proinflammatory processes and tissue repair in both adults and elderly patients is shedding valuable light on the implications of monocyte alterations in these critical biological processes. This expanding body of knowledge continues to enhance our understanding of the impact of monocyte diversity on health and disease.

## Monocyte continuum and spectrum approach

Recent research has unveiled that the traditionally defined monocyte subsets are not isolated entities but are interconnected throughout their differentiation stages. This growing interest in understanding their phenotypes has led to the utilization of various gating techniques to characterize and distinguish monocyte subsets, [20] Hijdra et al. for instance, employed a comprehensive approach, subdividing the monocyte plot into ten gates based on the expression of CD14 and CD16, to delve deeper into the intricacies of these subsets [21]. However, findings from studies in mice have suggested that monocyte subsets form a continuous spectrum of differentiation stages, challenging the notion that they can be entirely captured by conventional gating strategies [22]. Moreover, accumulating evidence suggests that the distinct nature of human monocyte subsets extends beyond the simple expression of CD14 and CD16 [14]. Patel et al. conducted in vivo deuterium labeling studies in humans, revealing that monocytogenesis involves the emergence of classical monocytes, which subsequently mature into intermediate and nonclassical monocytes. The timing of these transitions aligns with a physiological maturation cascade, providing substantial evidence that the traditionally defined monocyte subsets are indeed interconnected [14]. Furthermore, Hamers et al. employed advanced techniques such as mass cytometry and clustering algorithms to identify a total of eight monocyte subsets. These subsets included four within the classical subset, three within the nonclassical subset, and an intermediate population. While some markers, like SLAN, were specific to nonclassical monocytes, many markers were expressed by multiple subpopulations, albeit to varying degrees.

These findings collectively underscore the complexity and interrelated nature of monocyte subsets, challenging the previously held notion of discrete categorizations. This evolving understanding of monocyte heterogeneity enhances our ability to comprehend the intricate roles they play in health and disease, paving the way for more targeted research and potential therapeutic interventions.

## Monocytes subsets cytokines expression in response to various stimulation

Monocytes, traditionally categorized into three subsets based on their expression of CD14 (the lipopolysaccharide receptor) and CD16 (Fc gamma receptor III), have been the subject of extensive research [15]. However, as investigations have progressed, additional phenotypes have emerged based on gene and protein expression [15] patterns [19]. Notably, these monocyte subsets exhibit varying cytokine expression patterns depending on the stimuli, dosage, and kinetics they encounter. Each subset possesses its unique characteristics and responses to different immune challenges [19]. The nature of cytokine production by different monocyte subsets has indeed sparked debates in the scientific literature. To address this controversy, Ratnadeep et al. conducted experiments where they stimulated whole blood with LPS and quantified intracellular cytokines. Their findings shed light on the matter, revealing that ‘non-classical’ monocytes were the primary producers of the inflammatory cytokines IL-1β and TNF-α, whereas intermediate monocytes predominantly generated IL-10 [23].

In a similar vein, Wong et al. conducted a study comparing cytokine production among the three monocyte subsets after activation. Their results further enriched our understanding of monocyte functionality. They reported that classical monocytes produced the highest levels of cytokines like IL-6, IL-10, CCL2, and granulocyte colony-stimulating factor (G-CSF). On the contrary, the non-classical subset exhibited the highest levels of inflammatory cytokines such as TNF-α and IL-1β. Intermediate monocytes displayed cytokine production at an intermediate or lower level compared to the other subsets [12].

These findings underscore the intricate and context-dependent nature of cytokine production by monocyte subsets, emphasizing the need for a nuanced understanding of their roles in the immune response. Such insights have significant implications for our comprehension of immune regulation and could potentially inform targeted therapeutic strategies in the future.

## Monocyte mobilization and recruitment

The bone marrow stands as the primary hub for monocyte production, where these essential immune cells originate. The mobilization of monocytes from the bone marrow into the bloodstream is contingent upon the chemokine receptor CCR2 and its corresponding ligands, MCP-1 and MCP-3. These crucial ligands are typically produced by mesenchymal stem cells located near the bone marrow lumen or, in certain contexts, by B cells in peripheral tissues [24, 25]. It is worth highlighting that the release of monocytes into the bloodstream is not a constant process, even under steady-state conditions. Circadian rhythms, which orchestrate various biological processes throughout the day, also play a role in regulating monocyte mobilization [26]. This underscores the finely tuned nature of the immune system and its ability to adapt to different physiological states and challenges. When monocytes embark on their journey to sites of tissue injury or inflammation, their migration is facilitated by a gradient of chemoattractants, often referred to as chemokines. These chemokines act as signaling molecules, guiding monocytes precisely to where they are needed by binding to their cognate receptors on the monocyte surface [27]. This orchestration of immune cell movement is a vital component of the body’s response to infections, injuries, and other immune challenges, ensuring that immune cells are directed to the right place at the right time.

## Monocyte-macrophage differentiation

**Fig. 1:**
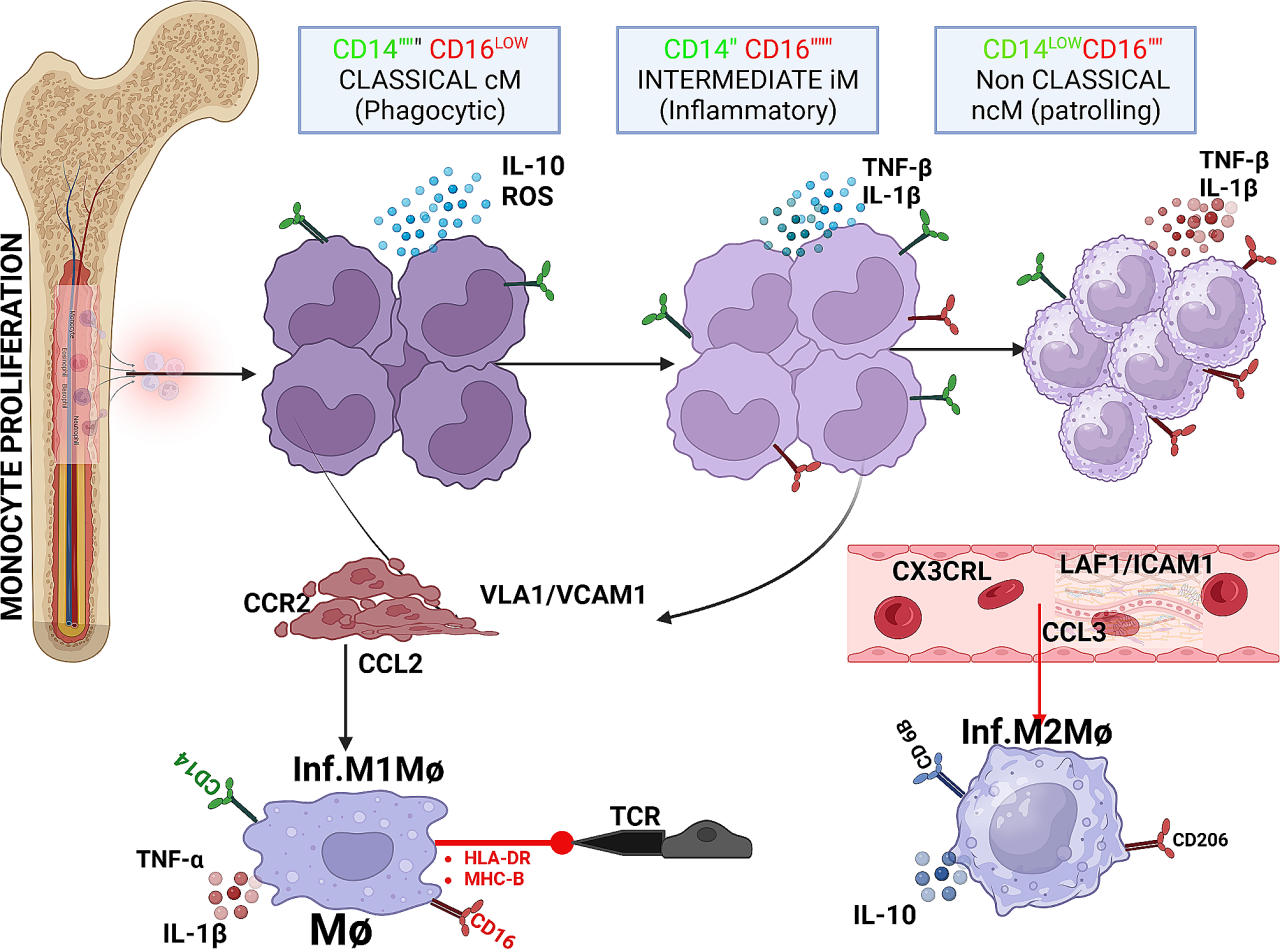
Human CD14 + + CD16- classical cM leave the bone marrow in a CC-chemokine receptor 2 (CCR2)-after fracture. During inflammation, classical and intermediate. MCs are tethered and invade tissue by interaction of complementary pair CCR2/CCL2(MCP1) or/and CCR5/CCL5(RANTES) in a VLA1/VCAM1 dependent manner. MCs then mature to 746 M1Mϕ in tissue and present self-antigen via MHC-I/II to TCR leading to TC activation. Non- classical MCs patrol the vessel wall and invade by interaction of complementary pair of 748 CX3CR1/CCL3 via LAF/ICAM1-dependent manner

Human monocytes possess remarkable plasticity, as they can differentiate into both macrophages (mo-Mac) and dendritic cells (mo-DC) under various in vitro culture conditions. Importantly, in vivo studies conducted in humans have demonstrated that monocytes and macrophages exhibit a high degree of plasticity and can undergo cross-differentiation into different subsets in response to changes in their microenvironment [28] (Fig. 1).

This flexibility underscores the dynamic nature of these immune cells and their capacity to adapt to diverse conditions. In some instances, unusual transitions of monocytes into macrophages have been observed. For example, in inflamed skeletal muscle or brain tissues, infiltrating Ly6C^+^ monocytes down-regulate Ly6C expression and acquire phenotypic features of anti-inflammatory monocytes. They exhibit functions akin to M2 macrophages, displaying an anti-inflammatory phenotype [29, 30]. This transition highlights the adaptability of monocytes in response to tissue-specific demands. Furthermore, Ly6Cmiddle monocytes can migrate to lymph nodes via the chemokine receptors CCR7 and CCR8, where they differentiate into dendritic cells [31]. This process demonstrates the versatility of monocytes in giving rise to different immune cell types, depending on their microenvironment. Research has also shown that under steady-state conditions, Ly6C^+^ monocytes are recruited to healthy lamina propria and differentiate into tissue-resident CX3CR1^high^ macrophages [32]. This highlights their role in maintaining tissue homeostasis.

Interestingly, in certain infections, such as Litomosoides sigmodontis infection, M2 macrophages are generated through the alternative activation of tissue-resident macrophages, rather than being recruited from monocytes [33]. This illustrates the diverse pathways through which M2 macrophages can arise in response to varying stimuli [34].

### Monocyte subsets alterations in the aging population

Monocytes occupy a pivotal role in a wide array of physiological processes, including phagocytosis, antigen presentation, orchestrating inflammatory responses, and contributing to tissue repair. Additionally, they have a substantial impact on age-related health conditions, such as atherosclerosis, inflammatory diseases, and Alzheimer’s disease [8, 35, 36]. A notable study by Sarra et al. in 2015 shed light on alterations in monocyte function linked to inflammation following hip fractures in elderly subjects, underscoring their relevance in age-related health issues [36]. Furthermore, it has been reported that classical monocytes in humans have the capability to undergo a transition to non-classical monocytes [14]. Recent research endeavors have identified specific changes in monocyte subsets concerning age and surgical interventions. Interestingly, while the classical monocytes (cM) and intermediate monocytes (iM) subsets showed no significant changes, the non-classical monocytes (ncM) subset exhibited a dramatic decrease before surgery, and this decrease was not reversed over time. This observation suggests a profound defect in the cell-adhesive properties of the ncM monocyte subset [14].

These findings underscore the multifaceted role of monocytes in both normal physiological processes and age-related health conditions. Understanding the alterations in monocyte subsets and their functional characteristics in response to various stimuli, such as surgery, is of critical importance in advancing our knowledge of immune responses and their implications for health and disease. Such insights may pave the way for novel therapeutic approaches aimed at mitigating the impact of age-related health conditions.

### Alteration of monocytes subsets according to lifetime

The aging process exerts a profound impact on the immune system, resulting in notable changes in immune cell numbers, phenotypes, and functions [37]. Chronic immune activation, a key feature associated with aging, contributes to two closely related phenomena: immunosenescence and inflammaging [38, 39]. Additionally, the distribution of human monocyte subsets in the bloodstream exhibits variations throughout one’s life, with distinct patterns observed during early life and after the age of 50 [40]. In newborns, there is a peak in the numbers of intermediate and non-classical monocytes, whereas classical monocyte populations reach their zenith in cord blood samples. Subsequently, these numbers decrease until the age of 8–13 years, only to rise again during adolescence, especially for the classical subset, and remain elevated in younger adults. However, after this phase, the numbers decrease once more, reaching a nadir between the ages of 30 and 50. Interestingly, during the earliest stages of life and after the age of 50, intermediate and non-classical monocytes may represent more advanced stages of maturation compared to the classical subset [14]. Furthermore, older individuals often exhibit elevated levels of pro-inflammatory cytokines, a phenomenon known as “inflam-aging” [41]. Studies have documented immunological changes following HF, including alterations in monocytes that lead to an inflammatory phenotype characterized by increased production of tumor necrosis factor-alpha (TNF-α) [8]. Phenotypic shifts in monocytes during fractures show similarities, such as an increased proportion of CD16^+^ monocytes accompanied by decreased expression of CX3CR1. A study by Vallet et al. observed decreased CX3CR1 expression and increased CCR2 expression, suggesting a robust turnover and recruitment of monocytes from the bone marrow, which may facilitate their migration to the fracture site. These findings collectively highlight the intricate relationship between aging, immune changes, and monocyte alterations, particularly in the context of age-related health conditions like hip fractures. Understanding these dynamics is crucial for developing strategies to mitigate the impact of age-related immune changes and improve the health and well-being of older individuals.

### Impact of aging alteration on monocyte subsets

The aging process has a substantial impact on the intermediate and non-classical monocyte subsets, resulting in significant changes in their numbers, phenotypes, and functional characteristics. A recent study by Cao et al. in 2022 shed light on this phenomenon, revealing that older adult exhibited a reduced frequency of Mo1 monocytes and increased frequencies and numbers of Mo0, Mo2, and Mo3 monocyte subsets when compared to young and middle-aged adults. This finding indicates a notable shift in monocyte subsets towards intermediate and non-classical phenotypes during the aging process [42]. Interestingly, the absolute number of CD16^+^ monocytes has been reported to increase in aged individuals, while the number of classical monocytes remains relatively stable [43]. Intermediate and non-classical monocytes are well-known for their significant production of pro-inflammatory cytokines, and the expansion of these populations may contribute to the chronic inflammation often observed in older individuals. Moreover, studies have indicated that both CD115 and TLR4 expression decrease with age on classical monocytes. This decline in expression may have important implications for the functioning and transcription of monocyte subsets and could potentially contribute to immune dysregulation and the increased susceptibility to infections often seen among older individuals [15] These findings highlight the intricate interplay between aging, changes in monocyte subsets, and the potential consequences for immune function and overall health in older adults. Understanding these dynamics is crucial for developing strategies to mitigate age-related immune changes and promote healthier aging.

### Alteration monocytes expression of cytokines and chemokine receptor markers in elderly

Numerous studies have delved into age-related changes in monocyte subsets, with a particular focus on the expression of cytokines and chemokine receptor markers [42, 44]. Numerous studies have delved into age-related changes in monocyte subsets, with a particular focus on the expression of cytokines and chemokine receptor markers [45]. Additionally, the reduced surface expression of certain proteins like HLA-DR, CX3CR1, and CD62L on monocytes in older adults may have implications for monocyte survival, adherence, and migration to sites of inflammation [30]. CD62L plays a pivotal role in monocyte rolling and adhesion to endothelial cells, and its downregulation is hypothesized to impair rolling and increase firm attachment of cells to vessel walls, a precursor to endothelial migration [45]. Research conducted by Seidler et al. in 2010 revealed significantly lower expression of HLA-DR on CD14^+^CD16^+^ monocytes in older individuals [30].

Furthermore, chemokine receptors CCR2 and CX3CR1 are differentially expressed on both monocyte subsets and have been implicated in their migration and function [22]. CD14^++^CD16^−^ monocytes express high levels of CCR2 and low levels of CX3CR1, while CD14^+^CD16^+^ monocytes express very low levels of CCR2 and high levels of CX3CR1. These findings suggest that not only does the absolute number of ‘non-classical’ CD14^+^CD16^+^ monocytes increase with age, but their phenotype also undergoes changes, resulting in lower expression of activation markers and chemokine receptors [46]. In the context of HF, which is associated with a state of inflammation, investigations have explored whether HF influences the expression of HLA-DR and CD11b in monocyte subsets. The findings indicate that there were no changes in the expression of HLA-DR in the intermediate (iM) and non-classical (ncM) subsets prior to surgery or during follow-up. However, significant increases were observed in the classical (cM) subset, but only at 6 weeks and 6 months after surgery [8]. These insights underscore the intricate relationship between age-related changes in monocyte subsets and their potential implications for immune function, inflammation, and specific health conditions like hip fractures. Understanding these dynamics can offer valuable insights into the mechanisms underlying age-related health issues and guide potential therapeutic strategies.

### Monocytes signatures specificity in diseases states and aging

In a cohort study conducted by Vallania and her colleagues, they identified notable gene expression patterns in monocyte subsets [47]. Specifically, they found that classical monocytes over-expressed 30 genes, while non-classical monocytes over-expressed 268 genes. To further characterize these subsets, they established gene signatures, each consisting of ten over-expressed genes, for both classical and non-classical monocytes. These signatures were rigorously validated using transcriptome profiles of 6661 sorted immune cells. This innovative approach enables the monitoring of changes in monocyte subset proportions associated with disease using these specific signatures and their corresponding scores, known as cMSS and ncMSS [48]. When examining the impact of aging on monocytes, recent research has yielded conflicting results.

However, with advanced techniques such as miRNA and monocyte transcription analysis, it becomes feasible to investigate individual monocyte subsets and formulate hypotheses about how aging may influence monocyte function at the transcriptional level. These dynamic changes in monocyte subsets may particularly manifest during the course of diseases, especially in the context of major systemic inflammation [20].

This enhanced understanding of the molecular underpinnings of monocyte subsets and their responses to aging and disease holds significant promise for unraveling the complexities of immune regulation and age-related health conditions.

### Monocytes transcriptional and functional alteration in aging

Age-related dysfunctions of the immune system encompass a spectrum of changes in the transcription, distribution, and function of cells that play pivotal roles in mediating communication between the innate and adaptive immune responses [15]. Among these immune cells, monocytes have garnered significant attention due to their transcriptional alterations during aging, raising global concerns about their functional changes in this context. These aging-related functional changes in monocytes have been associated with impaired phagocytic activity, diminished antigen presentation capacity, and alterations in cytokine production [49]. These changes in the early healing response, stemming from shifts in the innate immune system, can have downstream consequences on the adaptive immune system as a result of aging. Consequently, this may lead to impaired bone repair, delayed healing processes, and potentially the development of conditions such as osteonecrosis [50]. Understanding the intricate interplay between aging, immune dysfunctions, and their impact on the body’s ability to respond to injuries and challenges is of paramount importance for advancing our knowledge of age-related health conditions and developing strategies to promote healthy aging.

## Impact of monocyte alteration in elderly hip fracture

### Potential role of monocytes in bone resorption

Bone homeostasis is a highly intricate process, and its regulation extends beyond the musculoskeletal system to involve various biological systems of particular significance are the immune and skeletal systems, which share a common repertoire of regulatory molecules, including cytokines and signaling molecules. These molecules play indispensable roles in preserving and promoting bone health [22]. The interaction between bone metabolism and monocytes represents a dynamic and multifaceted process that exerts tight control over bone remodeling and healing. Monocytes, as integral components of the immune system, assume crucial responsibilities in maintaining and repairing bone tissue. They contribute significantly to the delicate equilibrium governing the processes of bone formation and resorption. This intricate interplay between the immune system, monocytes, and the skeletal system underscores the vital role of monocytes in bone health. It highlights their capacity to influence and modulate bone remodeling, emphasizing the importance of understanding these mechanisms for the development of strategies aimed at preserving and enhancing bone health.

### Osteoclasts, osteoblasts, osteocytes interrelation with monocytes in HF

Bone is a complex tissue sculpted by coordinated actions of three major cell types: the relatively short-lived bone-forming osteoblasts and bone-resorbing osteoclasts as well as the terminally differentiated osteocytes, which are former osteoblasts embedded within the mineralized matrix [13].

Activated monocytes play a pivotal role in promoting osteogenesis by transmitting pro-osteogenic signals to mesenchymal stem cells [51] Among these monocyte-derived cells, macrophages are particularly noteworthy as they actively support osteoblast differentiation and proliferation by releasing cytokines such as BMP-2, BMP-4, and TGF-β1, all of which are recognized regulators of osteoblastic cells [52]. Conversely, certain cytokines exert inhibitory effects on osteoblasts. For instance, TNF-α hinders osteoblast differentiation, and IL-1, TNF-α, and IFN-γ dampen collagen synthesis in osteoblasts. In contrast, IL-4 and IL-13 suppress prostaglandin synthesis in bone and act as chemoattractants for osteoblasts. Moreover, IL-4 fosters proliferation while inhibiting differentiation in osteoblastic cell lines [36]. These intricate interactions underline the significance of monocytes as a valuable model for studying bone-related diseases [53].

This makes monocytes a valid model for studying bone-related diseases [54]. Given that monocytes are implicated in both inflammation and bone resorption, they emerge as central regulators of bone tissue [55]. Osteoporotic hip fractures, which are linked to high morbidity and mortality rates, contribute significantly to healthcare expenditures [56].

The role of monocytes in the complex interplay between inflammation and bone health underscores their importance in understanding and addressing bone-related disorders, making them a compelling focus of research in this field.

### Monocytes response and function in post hip fracture

**Fig. 2:**
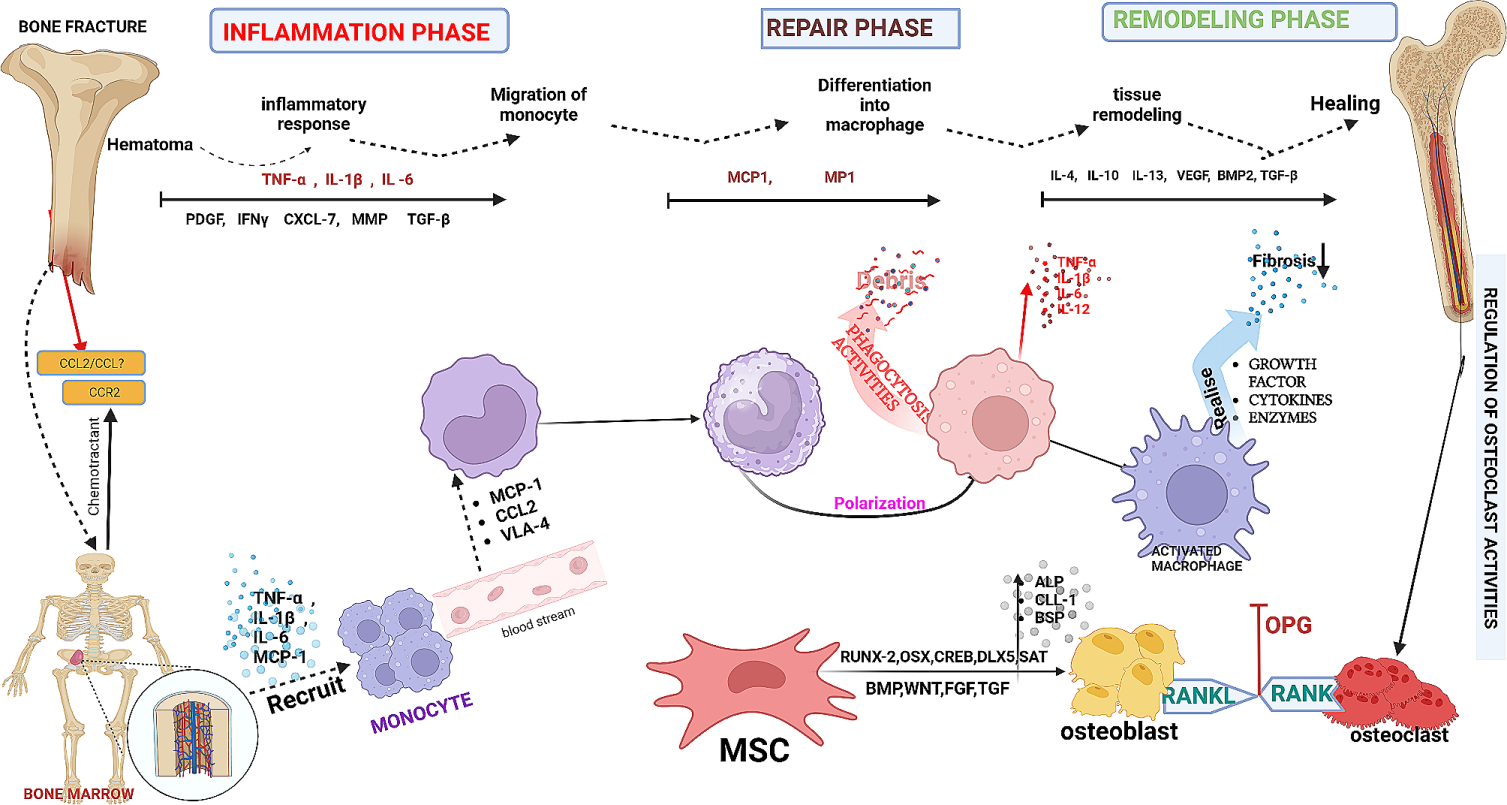
Schematic representation of the possible recruitment and regulatory functions of monocyte. The entire healing process of an occurring fracture can be broadly divided into three main phases, i.e., the inflammatory phase, repair phase and the tissue regeneration phase, during which monocyte participate and regulate subsequent fracture repair. Fractures lead to the release of cellular signaling of chemokine to bone marrow, which triggering the release of monocyte from bone marrow through cellular signaling. During the inflammatory phase, inflammatory mediator cytokine and chemokine through chemoattractant recruit monocytes from the bone marrow to the site of injury and participate in the immune response by phagocytosing cellular debris and secreting proinflammatory cytokines (e.g., TNF, IL-1β, IL-6 and IL-12). Differentiate into M1 macrophages promote tissue regeneration in the early and middle stages without enhancing matrix mineralization. activated macrophage release growth factors, cytokines, enzyme to stabilize the regeneration process in the late repair phase, M2 (anti-inflammatory) macrophages release regenerative cytokines such as IL-10, TGF-β, BMP-2 and VEGF to establish an anti-inflammatory environment which promotes osteoclast genesis and bone healing

Fractures healing is a highly intricate physiological process that necessitates the precise coordination of numerous cell types and signaling pathways (Fig. 2). A pivotal facet of this process hinges on the involvement of inflammatory factors that are secreted by immune cells. These factors assume indispensable roles in governing the recruitment, proliferation, differentiation, and activation of hematopoietic and mesenchymal cells, all of which are critical for the successful healing of bone fractures [57]. Inflammation stands out as a crucial mediator, playing a central role in initiating the repair process and masterminding the intricate interactions among diverse cell types. These orchestrated interactions are paramount for promoting efficient bone regeneration. However, the mechanisms by which senescent cells and the monocyte potentially alter bone remodeling are incompletely understood, leaving a significant gap in knowledge. Understanding the intricate web of inflammatory responses and their impact on bone healing is pivotal for advancing our knowledge of this complex process and developing strategies to optimize bone fracture recovery.

### Cellular senescent in the bone microenvironment of elderly

Cellular senescence refers to the stable state of cell cycle arrest, with senescent cells emerging in the early stages of embryonic development and accumulating with age [58]. Senescent cells adversely affect tissues by secreting excessive inflammatory cytokines, chemokines, oxidative stress-related proteins, growth factors, and proteases, leading to what is known as the senescence-associated secretory phenotype (SASP) [59]. During the aging process, senescent cells tend to aggregate in the skeletal system and induce chronic inflammation by releasing SASP factors [60].

Throughout life, the skeleton is continuously turned over via a self-renewal process termed bone “remodeling” whereby old damaged bone that has accumulated microfracture fatigue is resorbed by osteoclasts and under normal conditions is replaced with an equal amount of new bone by osteoblasts. These actions are coordinated by osteocytes–the “master regulators” of bone remodeling that coordinate the actions of osteoblasts and osteoclasts through [61]. With aging, however, these actions are disrupted leading to decreased bone formation (less new bone laid down) relative to resorption (more old bone removed), and ultimately negative bone balance. Over prolonged periods of time, these adverse events cause dramatic bone loss, resulting in osteoporosis – a very common, devastating skeletal fragility syndrome causing > 9 million worldwide fractures annually [62]. Age-related senescence effects in bone where they may contribute to impaired osteoblastprogenitor cell function, defective bone formation, andincreased osteoclastogenesis [63].

### The impact of monocytes in the elderly HF healing

In elderly individuals, the process of healing HF becomes notably challenging due to several key factors, including the age-related decline in immune function and alterations in monocyte functionality [64]. Age-related changes can have a significant impact on the recruitment and mobilization of monocytes to the injury site, potentially compromising their ability to respond to chemokines and effectively migrate to the fracture site [65]. This, in turn, may result in delayed or reduced monocyte infiltration, thereby affecting the timely initiation of the healing process. Moreover, age-related alterations can disrupt the balance and proportions of monocyte subsets [66]. Studies have consistently indicated an increased proportion of non-classical monocytes (characterized by the CD14^+^CD16^++^ subset) in the elderly population. These non-classical monocytes possess a diminished capacity to phagocytose debris and contribute to tissue repair [8]. Notably, recent research by Sebastian et al. highlighted how this imbalance in monocyte subsets can indeed impact the overall healing process of hip fractures in the elderly. Furthermore, the presence of a chronic inflammatory state can be detrimental to the healing process, impairing proper wound healing and tissue regeneration. Sebastian Seidler et al. recently demonstrated that an imbalance in monocyte subsets may impact the overall healing process of hip fractures in the elderly. A chronic inflammatory state can be detrimental to the healing process, impairing proper wound healing and tissue regeneration [67]. Talibah et al. also underscored that activated monocytes within this chronic inflammatory state may contribute to the excessive production of pro-inflammatory cytokines, such as interleukin-6 (IL-6) and tumor necrosis factor-alpha (TNF-α). This, in turn, hinders the resolution of inflammation and impedes the orderly progression of tissue healing [15].

The intricate relationship between aging, altered monocyte function, and the inflammatory response in the context of hip fractures among the elderly underscores the multifaceted challenges in achieving effective and timely healing in this vulnerable population. Understanding these complexities are crucial for developing targeted therapeutic strategies to improve outcomes in elderly individuals with hip fractures.

## Promising monocytes subsets and biomaterials in hip bone fracture healing

In recent years, the significance of the intricate interplay between the immune and skeletal systems has come to the forefront, giving rise to the interdisciplinary field of osteoimmunology, which delves into the intricate interactions between these two systems [68]. The consolidation of critical bone injuries is still an emerging clinical problem, because extensive tissue losses resulting from traumatic or extensive fractures generally have biological and physiological limitations that impair the adequate bone tissue repair [69].

Generally, bone tissue has high regenerative capacity, but, when it comes to areas with considerable extensions, this capacity is compromised, resulting in delayed consolidation [70]. Regenerative medicine faces a profound challenge in developing novel strategies aimed at enhancing bone health and improving the quality of life for patients grappling with bone injuries and diseases [71]. The process of bone regeneration is highly complex, marked by the meticulous coordination of various biological events. Immune cells, particularly monocytes, assume a prominent role as they produce soluble factors that robustly stimulate the expression of osteogenic genes and facilitate the differentiation of mesenchymal stem cells (MSCs). Monocytes, functioning as crucial first responders to tissue injury, are integral to the success of tissue regeneration. In the context of bone injuries, monocytes and macrophages play pivotal roles in maintaining bone homeostasis and advancing fracture repair by modulating the acute inflammatory response, generating growth factors, and fostering the differentiation of mesenchymal progenitors [72].

Prognostic markers for HF have been identified, including an upsurge in pro-inflammatory cytokines such as IL-6 and TNF-α, alongside a few established biomarkers like C-reactive protein and procalcitonin. Comprehending the intricacies of osteoimmunology in the context of fracture healing holds the potential to yield innovative treatment strategies and ultimately enhance prognoses for individuals with bone injuries [73]. In line with this, recent advancements have significantly improved our understanding of the immune response in wound healing, driving a growing interest in the development of scaffolds with immune-modulating capabilities [74]. Remarkably, recent studies have revealed that various biomaterials can exert a profound influence on macrophage function in vivo, ultimately shaping macrophage polarization [75].

### Therapeutic of monocytes in biomaterial for bone repair flowing fracture

With the developments in the technology of bone tissue engineering and orthopedics have been ongoing. Artificial bone can now be customized and massproduced. Bone tissue engineering has enabled the development of a bone surrogate, which has the ability to repair bone defects following tissue engineering principles and methods [76]. The ultimate goal of bone tissue engineering is to regenerate damaged or defective The immune response must be carefully considered in the material design in order to avoid unwanted cell recruitment/attachment, heightened secretion of inflammatory cytokines, fibrous encapsulation, or chronic inflammation [77].

Therapeutic of Monocytes/Macrophages Combined with Biomaterials in Bone Regeneration Monocytes have shown remarkable plasticity and have been recognized not only as proinflammatory cells but also as reparative cells [78, 79]. (McLaughlin S, 2022) As we mentioned earlier in this review, the characterization and functionality of monocyte subsets are still being studied, allowing for potential modifications in therapeutic and pharmacological approaches not only for hip fracture healing but also in controlling inflamm-aging. Monocytes and macrophages play a crucial role in the immune response to biomaterials, directly affecting the biocompatibility of biomedical devices and the outcome of successful implantation. In recent years, many studies have focused on promoting macrophage polarization to an anti-inflammatory phenotype to accelerate the process of wound healing and bone regeneration [80–82]. In addition, Li et al. showed that skeletal stem cells delivered using microgels had a more significant preventive effect on macrophage activation at osteoarthritis sites [83]. Scaffold materials can regulate macrophage phenotypes through their physicochemical properties, enabling them to secrete appropriate cytokines and angiogenic growth factors at different stages to guide the recruitment, proliferation, and differentiation of vascular endothelial cells and other cells, thereby promoting angiogenesis [84, 85]. In the field of bone regeneration, research on scaffold materials has attracted much attention, and Stepanova et al. introduced a drug-loaded 3D printed polymeric scaffold that can be used for localized antimicrobial or anti-inflammatory therapy for bone regeneration [86]. In addition, Xiong et al. showed that hydroxyapatite scaffolds with a pore size of 600 μm could promote macrophage M2 polarization, thereby modulating the immune microenvironment and enhancing bone regeneration [87]. Total hip arthroplasty (THA) now utilizes ceramic-on-ceramic (cer-cer) or metal-on-metal (met-met) coupling, which is considered the bearing of a new generation with improved performance compared to previous polyethylene bearings. While ceramic-on-ceramic is universally considered highly biocompatible, met-met coupling can induce hypersensitivity correlated to an immunological response. This highlights the importance of understanding and considering the immune response in choosing appropriate materials for orthopedic implants.

## Conclusions and perspectives

In conclusion Osteoimmunology research studies have provided valuable insights into the characteristics, presence, and potential function of monocyte subsets under homeostatic and inflammatory conditions. Moving forward, the challenge lies in developing strategies to effectively track the fate of specific subsets in therapeutic contexts to better understand the potential importance of monocyte conversion during elderly HF and identify targets for therapeutic use. In this review, we highlighted Aging significant impact on the immune system of elderly individuals, leading to comprehensive changes in both innate and adaptive features of monocyte mechanisms. These age- related alterations in cellular processes are evident during fracture healing and are accompanied by changes in the regulation of critical genes involved in bone fracture healing between older and younger patients after severe bone fractures. we have summarized several studies that highlight the crucial impact of monocytes which exhibit diverse functional in normal activities, including migration, pattern recognition, scavenging, phagocytosis, antigen presentation, and interaction with lymphocytes. Their flexible nature needs to be harnessed and engineered to provide biomaterials for the regeneration process and bone tissue healing. Nano-systems have shown the potential to enhance bone regeneration, a complex process requiring the interplay between immune and skeletal cells. Activated monocytes can communicate pro-osteogenic signals to mesenchymal stem cells and promote osteogenesis, making monocyte activation a promising strategy to improve bone regeneration [52]. Taken together, further efforts are required to gain an in-depth insight into a significant value of monocytes subsets alteration in aging-hip fractures and would provide new potential molecular understanding for research and development of pharmacological and clinical therapies for values aging patients with hip fracture.

### Limitations

The monocyte research is relatively limited and may not be able to provide a very comprehensive overview of other research results related to HF and other bones fractures. many literatures highlighted research accomplished in recently years, but some aspects, especially the function, nomenclates of monocytes subtypes, have changed to oppose the previous classical concepts. Therefore, some results from the latest research seem contradictory each other’s. However, we believe that recent literature is more valuable for reference; thus, readers need to understand the limitations of this review in terms of time and source.

## Data Availability

No datasets were generated or analysed during the current study.
